# An experimental study of deformation and fracture characteristics of shale with pore-water pressure and under triaxial cyclic loading

**DOI:** 10.1098/rsos.180670

**Published:** 2018-08-22

**Authors:** Changbao Jiang, Tianyu Lu, Dongming Zhang, Guangzhi Li, Minke Duan, Yufei Chen, Chaosheng Liu

**Affiliations:** 1 State Key Laboratory of Coal Mine Disaster Dynamics and Control, Chongqing University, Chongqing 400030, People's Republic of China; 2 College of Resource and Environmental Science, Chongqing University, Chongqing 400030, People's Republic of China; 3 CCTEG Chongqing Engineering Co. Ltd, Chongqing 400016, People’s Republic of China

**Keywords:** shale, pore-water pressure, cyclic loading, deformation, computed tomography scan, failure mechanism

## Abstract

The deformation and fracture characteristics of shale in the Changning-Xingwen region were experimentally studied under triaxial cyclic loading with a controlled pore-water pressure. An RLW-2000M microcomputer-controlled coal-rock rheometer was used in the State key Laboratory of coal mine disaster dynamics and control in Chongqing University. These experimental results have indicated the following. (i) The shale softened after being saturated with water, while its failure strength decreased with the increase of axial strain. (ii) A complete cyclic loading–unloading process can be divided into four stages under the coupling action of axial cyclic loading and pore-water pressure; namely the slow or accelerated increasing of strain in the loading stage, and the slow or accelerated decreasing of strain in the unloading stage. (iii) The axial plastic deformation characteristics were similar when pore-water pressures were set to 2, 6 and 10 MPa. Nevertheless, the shale softened ostensibly and fatigue damage occurred during the circulation process when the pore-water pressure was set to 14 MPa. (iv) It has been observed that the mean strain and strain amplitude under axial cyclic are positively correlated with pore-water pressure, while the elastic modulus is negatively correlated with pore-water pressure. As the cycle progresses, the trends in these parameters vary, which indicates that the deformation and elastic characteristics of shale are controlled by pore-water pressure and cyclic loading conditions. (v) Evidenced via triaxial compression tests, it was predominantly shear failure that occurred in the shale specimens. In addition, axial cyclic loading caused the shale to generate complex secondary fractures, resulting in the specimens cracking along the bedding plane due to the effect of pore-water pressure. This study provides valuable insight into the understanding of the deformation and failure mechanisms of shale under complicated stress conditions.

## 1. Introduction

As an important successor of conventional energy sources, shale gas is one of the key new energy resources that will be developed over the coming era due to its widespread distribution and relatively clean combustion [1,2]. China has an abundance of shale gas resources which has led to the conduction of large-scale exploration, the findings of which indicate that the Sichuan Basin has a particularly substantial capacity for industrial production, due to its superior development prospects in comparison to other regions in China [3–5]. Thus, it is the most auspicious and significant area for the exploration of shale gas and development in China, and experimental study of shale in this area is of vast engineering and practical significance.

Shale gas constantly accumulates in rock fractures, causing abnormally high pressure in this area as a result of the joint action of high-pressure shale gas coupled with ground stress in the seam. Therefore, the surrounding rock is always under high confining stress. Hydraulic fracturing is the key technology in terms of the development of shale gas. After the reservoir has been reformed by the hydraulic fracturing technology, a fractured net is formed with artificial fractures penetrating the natural fractures [6,7]. After the shale gas field has been mined for a period of time, there is also typically a decrease in the production of gas, which leads to an urgent need for repeated fracturing. When fully completed, the shale gas pressure and fracturing fluid pressure decrease with the back discharge of the high-pressure fracturing fluid. Therefore, during the process of shale gas field development, shale reservoirs are always subject to the combination of cyclic loading and water pressure, which is integral to the stress–permeability coupling problem.

In recent years, scholars have carried out a number of studies on rocks which have been subjected to cyclic loading and unloading conditions. Jiang *et al*. [8] researched the seepage properties, acoustic emission characteristics and energy dissipation of coal. Tutuncu *et al*. [9] have pointed out that the characteristics of the stress–strain hysteresis curve are related to the frequency, the strain amplitude, the property of the saturated fluid for applying the load, and rock attenuation characteristics. Liang *et al*. [10] conducted a series of experiments on the mechanical properties of salt rock and studied the influence of strain rate. Liu & Liu [11] found that confining pressure and joint inclination greatly influenced the dynamic deformation and failure mechanism of rock, including the generation and extension of weak surface and micro-cracks. Fuenkajorn & Phueakphum [12] studied the effect of cyclic loading on uniaxial compressive strength, elastic modulus and irreversible deformation of rock salt. Bagde & Petroš [13] investigated the effect of load frequency and amplitude on fatigue failure strength and elastic modulus. Wei *et al*. [14,15] analysed elastic characteristics and fracture characteristics when subjected to confining pressure and cyclic loading.

At present, in the field of research pertaining to the influence of pore-water pressure on the mechanical properties and the deformation and failure characteristics of rock, studies have primarily been carried out using laboratory experiments and numerical simulations [16–23]. Hamiel *et al*. [18] analysed rock expansion, nonlinear characteristics and changes of pore-water pressure under shear and established the model used to demonstrate the sliding damage caused by pore-water pressure changes. The research of Xie & Shao [19] established that pore water caused the transient plastic deformation of the specimens and resulted in the decrease of yield stress and failure strength. Spencer *et al*. [20] pointed out that with the increase of pore-water pressure, the bulk modulus decreased, but Poisson's ratio increased when under high temperature and high pressure. In addition, Bruno & Nakagawa [21] found through the observations of field tests that the effects of pore-water pressure on crack propagation and penetration are bidirectional. Xu *et al*. [22,23] discussed the effect of pore-water pressure on the deformation characteristics of sandstone through cyclic loading–unloading experiments with the same confining pressure.

The above research results have vital referential value for studying mechanical, deformation and pore-water pressure characteristics of rock. However, while most of these studies refer to sandstone and salt rock, there are few reports which research the related topic of shale. The existing shale-related experimental studies [24–26] only analyse its brittleness and rupture characteristics, yet the interactions of cyclic loading, water pressure, and the geological parameters of shale reservoirs are not covered. Thus there are certain limitations to the previous research. Based on the *in situ* stress conditions of the shale in the Changning-Xingwen area, this paper studies the deformation and fracture characteristics of shale when subjected to pore-water pressure and triaxial cyclic loading through experiments, and also discusses the coupling mechanism between pore-water pressure and cyclic loading. Furthermore, this paper is of vast practical significance for correctly understanding the failure mechanism of shale under complicated stress conditions as well as the hydraulic fracturing effect of shale reservoirs.

## 2. Experimental set-up

### 2.1. Preparation of shale samples

**Figure 1.**
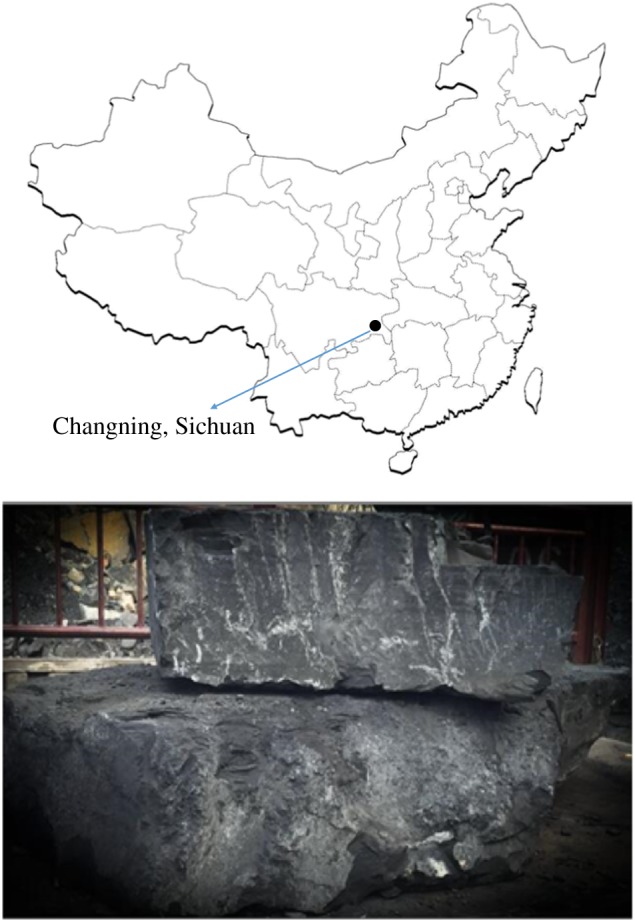
The location and a schematic diagram of shale sampling.

The shale used in this experiment was taken from the outcrop section of Shuanghe Town, Changning County, Yibin City, Sichuan Province. Intrinsic to the black shale of the Lower Silurian Longmaxi Formation, this site is located in the Changning-Xingwen shale gas producing area in southeastern Sichuan Province, as shown in figure 1. The buried depth of the shale reservoir is approximately 2300–3200 m, while the thickness is 250–300 m [27]. The lithology is black, deep, carbonaceous shale [28], which has a biological structure and an extremely thin bedding structure and contains fossil bedding surface as its weak surface. The content of total organic carbon was 10.24–11.26% in the prophase test. In order to reduce the variability in the sample, all the specimens have been taken from the same block of intact shale and drilled in strict accordance with the requirements of the International Rock Mechanics Program. A total of 10 standard cylinder specimens were obtained with a diameter of 50 mm, a height of 100 mm, and were perpendicularly axial to the bedding plane.

### 2.2. Experimental apparatus

**Figure 2.**
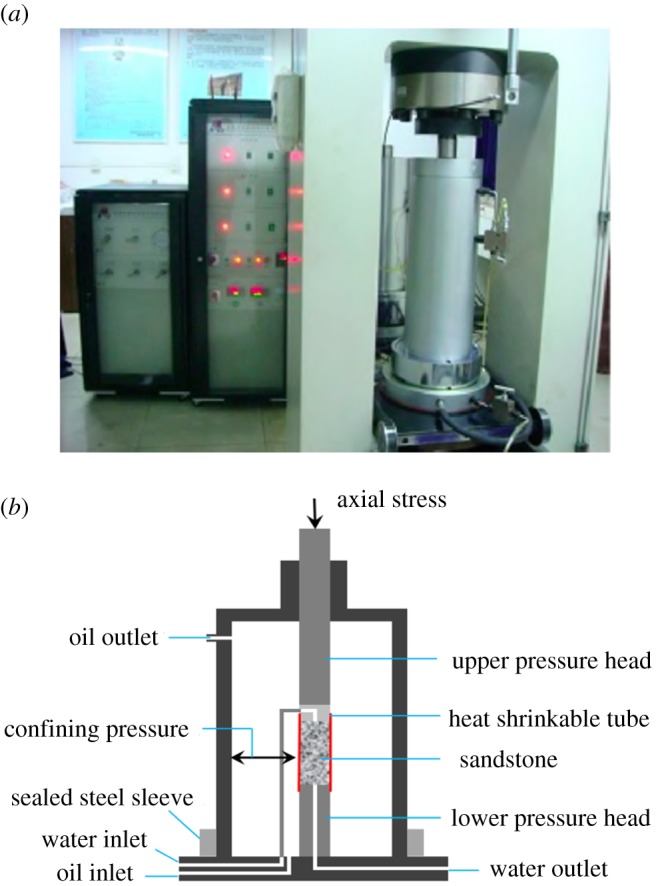
Experimental apparatus: (*a*) control testing system; (*b*) scheme of the loading device.

The equipment used in this experiment is a RLW-2000M microcomputer-controlled coal–rock rheometer from Chongqing University. This is an experimental device for the study of the rheological properties of coal and rock when subjected to various conditions. It can automatically perform uniaxial compression tests, triaxial compression tests, cyclic loading and rheological tests on coal or rock. The maximum axial load can reach up to 2000 kN. The maximum confining pressure and the maximum pore-water pressure can reach 60 MPa and 50 MPa, respectively, as shown in figure 2.

### 2.3. Testing scheme

**Figure 3.**
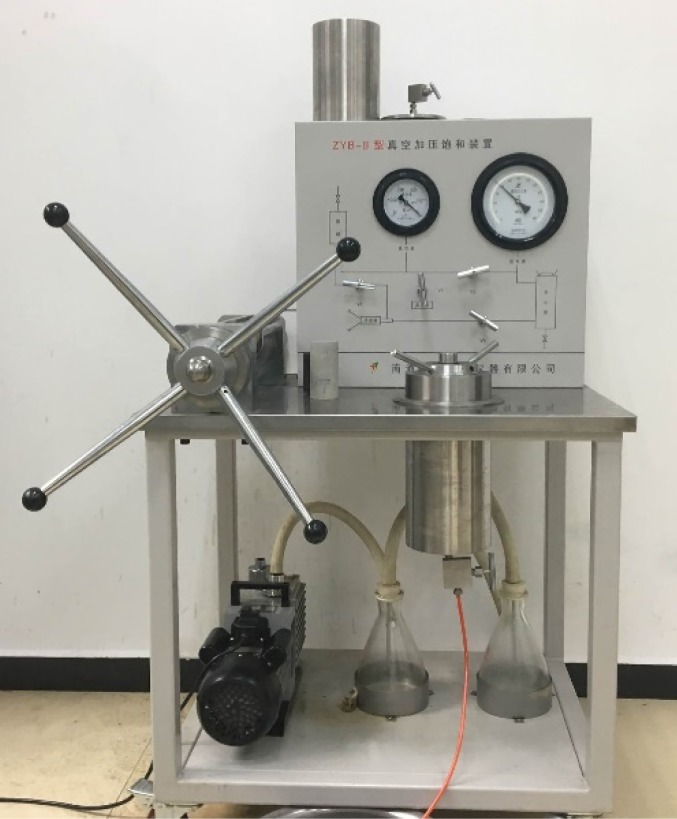
Water-saturated apparatus.

Because pore-water pressure is involved in this experiment, preparing fully saturated shale specimens is necessary. Firstly, the specimens were placed in an oven for 5 h, with a constant temperature controlled at 105°C. Then, the dried shale was placed in the water-saturated apparatus, as shown in figure 3. It was first evacuated, then distilled water was injected into the cavity. Finally, the water pressure was loaded in order to ensure that the shale was under a certain water pressure. During the next 24 h, continuous attention was paid to the changes in water pressure inside the chamber. If it was found that the water pressure decreased, the water pressure would then be increased to the set value until it remained constant. After the saturation stage was complete, shale specimens with no cracks on the outer surface were selected. The basic parameters of some specimens are shown in table 1, wherein the degree of saturation of the shale is 1.72–1.84%.

**Table 1.** Basic parameters of select shale specimens.

serial number	diameter (mm)	length (mm)	drying mass (g)	saturated mass (g)	degree of saturation (%)	drying density (g cm^−3^)	saturated density (g cm^−3^)
Z-01	48.90	100.02	453.3	461.4	1.79	2.413	2.456
Z-02	49.04	99.88	451.6	459.5	1.75	2.394	2.436
Z-03	49.12	99.96	459.0	466.8	1.70	2.423	2.464
Z-04	48.96	99.92	453.2	461.2	1.77	2.409	2.452
Z-05	49.02	99.90	453.4	461.1	1.70	2.405	2.446
Z-06	49.04	99.86	452.5	460.4	1.75	2.399	2.441
Z-07	48.94	99.98	452.1	460.4	1.84	2.404	2.448
Z-08	49.02	99.88	454.3	462.5	1.81	2.410	2.454
Z-09	49.04	99.92	453.5	461.3	1.72	2.403	2.444

The vertical crustal stress of the shale reservoir ranged between 56 and 66 MPa [27]. The following equation was used to estimating the horizontal stress based on the vertical stress and the depth [29]:

2.1 $$\frac{100}{H}+0.3\leq \frac{\sigma_{h,\mathrm{av}}}{\sigma_{v}}\leq \frac{1500}{H}+0.5,$$

where *H* is the depth, *σ*_h,av_ is the horizontal stress, *σ*_v_ is the vertical stress. The calculation results show that the horizontal stress is found to be between 20 and 66 MPa. Considering the substantive condition of the apparatus, the confining pressure of this experiment is set at 20 MPa. In the first test group, a triaxial failure experiment on the saturated shale specimens was carried out. The specimen was installed in the equipment, preloading the confining pressure to 20 MPa. The test would not be stopped until the specimen broke down with the loading of axial pressure by means of strain control. The specimen was then uninstalled, and the next set of experiments were carried out accordingly.

**Figure 4.**
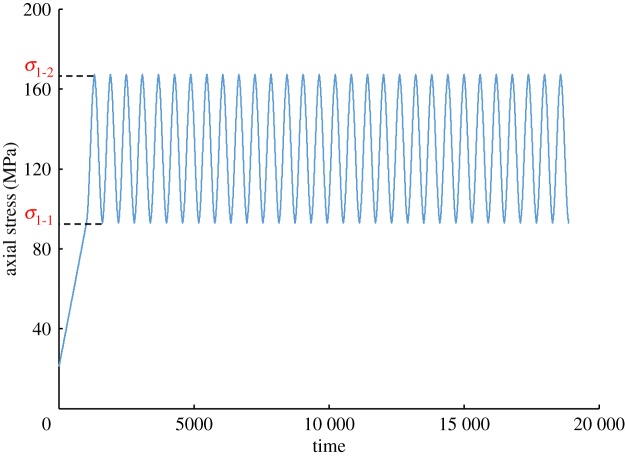
Curve of axial stress–time.

For the second group, a triaxial cyclic loading experiment with different pore-water pressures was performed. After the installation of the test specimen, the confining pressure was applied to 20 MPa, and the axial pressure was set to 40 kN. In this paper, the confining pressure was not directly loaded to 20 MPa, but was loaded step by step in accordance with the axial stress in order to ensure that the sample was in a triaxial equal pressure state. According to the experimental requirements, the pore-water pressure was set to 2 MPa, in order to maintain the pre-loading state until the water flowed from the outlet of the loading device. The outlet valve was then closed, and the axial load was applied then proceeded to use auto-control in order to carry out the experiment. Referring to the measured triaxial compressive strength of the saturated shale, the pressure of the equal amplitude cycle was set as 90–161 MPa (50–90% of axial failure pressure), as shown in figure 4. The cosine loading method was adopted using a frequency of 0.005 Hz (the time of one cycle is 200 s) and a cycle time numbering 30. The experiment would be stopped if the specimen broke down during the cycles, or persistently loaded until the specimen broke down after the cycles. After the test was finished, firstly the pore-water pressure was unloaded, followed by the unloading of the axial pressure. Finally, the confining pressure was released, and the specimen was removed. The next set of experiments was continued by repeating the above steps, only changing the pore-water pressure to 2 MPa, 6 MPa, 10 MPa, 14 MPa. After all experiments were completed, the damaged shale was scanned by computed tomography, of which the resolution is 0.4 mm.

## 3. Experimental results and study

### 3.1. The stress–strain curve of triaxial monotonic compression

**Figure 5.**
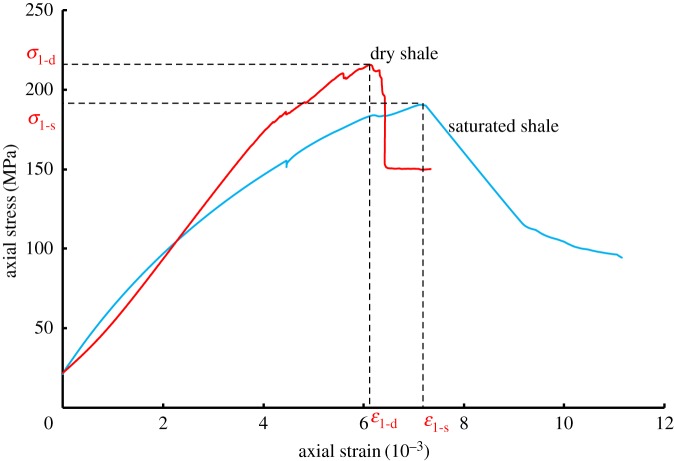
Triaxial stress–strain curve of shale under a confining pressure of 20 MPa.

Figure 5 shows the axial stress–axial strain curves of the saturated shale and dry shale under triaxial monotonic compression. The stress–strain curve of the dry shale behaves like a typical rock. In the initial stage of loading, the original open-type structure surface and micro-cracks in shale gradually closed, and the curve showed a concave shape. As the stress increased, the curve appeared approximately linear, exhibiting a stage of elastic deformation. When the stress increased to 160 MPa, the specimen exhibited stage failure, and the rock began to change from an elastic to a plastic state. Moreover, the rupture continued to develop until the specimen became completely destroyed. In comparison to previous studies [30,31], the failure strength in this study was found to be much larger.

However, the stress–strain curve of a saturated specimen exhibited different characteristics. As shown in figure 5, the curve showed a convex shape in the initial stage of loading, which indicates that the process of pore compaction did not occur. This is because the shale specimen had been soaked in a high-pressure solution for 24 h through a saturation process wherein the original pores and open-type structure surface had been compacted. With the increase of stress, the slope of the curve decreased slowly, and the specimen gradually reached the yielding stage. When approaching a peak, the specimen began to exhibit a periodic failure. After reaching the peak value, it was found that there was a significant reduction in stress, which is likely due to the destruction of the internal structure of the specimen. Comparisons showed that pore water would soften the shale, leading to a decrease in the compressive strength and an increase in the corresponding axial strain at the failure point. Therefore, the influence of pore water should be taken into consideration in engineering practices.

### 3.2. The stress–strain curve of triaxial cyclic compression

**Figure 6.**
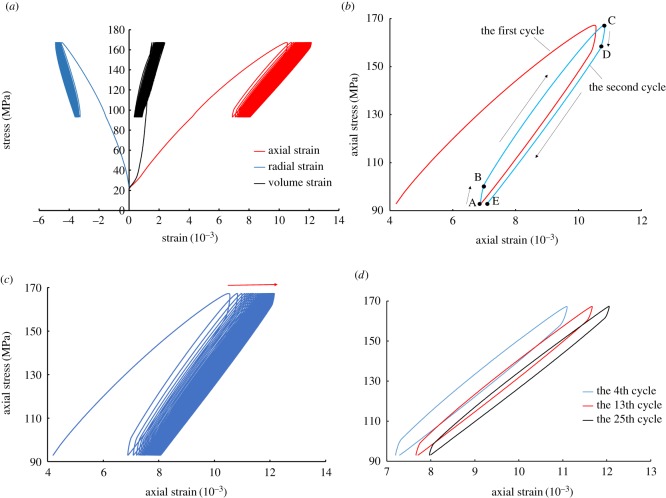
Stress–strain curve of shale under a pore-water pressure of 6 MPa: (*a*) complete stress–strain curve; (*b*) the previous two cycles; (*c*) the development direction of axial strain; (*d*) the 4th, 13th, and 25th cycles.

Figure 6*a* demonstrates the stress–strain curve of a shale specimen under triaxial cyclic loading at a pore-water pressure of 6 MPa. As shown in figure 6, during the initial stage of the monotonic loading, no pore packing of shale was observed in the curve. During the loading process, the axial plastic strain increased gradually throughout the course of the cycle, but the amplitude of the increase became progressively lower. Moreover, the absolute value of the radial strain increased, with positive volumetric strain, indicating that the specimen was in a state of overall compression relative to the initial stage.

As shown in figure 6*b*, during the stage of cyclic loading–unloading, barring the first cycle, each cycle was divided into four stages starting from the second cycle. (i) In the first stage of AB, due to the influence of the experimental machine and the initial pore-water pressure, the strain lags behind the stress, after which the stress changes from decreasing to increasing. This manifests as a slow increase of strain with an increase of stress. This was presumably due to the occurrence of plastic deformation before the cyclic loading began. Additionally, the specimen was under a lower load, wherein the influence of the pore-water pressure increased, and the viscous force of the rock enhanced [32], strengthening the ability of the rock to hinder deformation. (ii) In the second stage of BC, with the increase of axial load, micro-cracks and pores in the specimen were re-compacted, causing the specimen to show micro-elastic characteristics with an increasing axial deformation rate. (iii) In the third stage of CD, the specimen was in a high-pressure state during the initial unloading stage, and the distance between the mineral particles became closer [33]. Furthermore, a higher normal force on the contact surface led to an increased friction between the interfaces, resulting in the deformation rate being relatively slow. (iv) In the fourth stage of DE, due to the gradual decrease of axial load applied to the rock at the later unloading stage, the mineral particles in the rock were separated, and the micro-cracks reopened. This caused the pore water to refill, weakening the interfacial friction and speeding up the decrease of deformation.

As shown in figure 6*c*, as the number of cycles increased, the loading and unloading curves began to shift to the right, whereas the plastic deformation decreased and tended to be stable. Furthermore, the stress–strain curve became increasingly intense, and thereafter the shale entered the stable deformation stage. Figure 6*d* corresponds to the loading–unloading stress–strain curves of the 4th, 13th and 25th cycles. The comparison analysis shows that the unclosed degree of the hysteresis loops was higher during the initial cycles. Throughout the cyclic loading–unloading processes, the shale gradually compacted, thus the stiffness would be expected to increase. Moreover, it was found that the loading curve gradually moved closer to the unloading curve. However, the hysteresis loop was not completely closed, which is likely due to the existence of pore-water pressure. Thus, the micro-cracks and the weak surface within the specimen could not theoretically be completely closed. There is always a viscous effect inside the shale, and the plastic deformation would not be reduced to zero.

**Figure 7.**
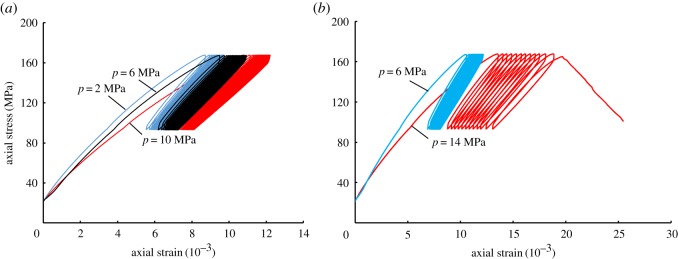
Stress–strain curves under different pore-water pressures: (*a*) the pore-water pressures are 2, 6 and 10 MPa; (*b*) the pore-water pressures are 6 and 14 MPa.

As shown in figure 7*a*, when the pore water pressure was set to 2, 6 and 10 MPa, the slope of the stress–strain curve decreased with the increase of pore-water pressure. According to the Terzaghi effective stress principle, the effective stress decreases with the increase of the pore-water pressure, so the elastic modulus of the shale is expected to decrease correspondingly during the monotonic loading stage, resulting in a decline in the slope of the curve. However, the stress–strain curves show that the plastic deformation was similar, even under different pore-water pressures during the process of cyclic loading–unloading, and no fatigue failure occurred in the processes.

However, when the pore-water pressure was set to 14 MPa, the axial deformation behaviour of the shale varied greatly. As shown in figure 7*b*, after entering the cyclic loading–unloading stage, except for the larger residual (plastic) strain of the first cycle, the residual strain of each cycle did not obviously decrease, but rather continued to increase. Furthermore, the hysteresis loops formed were not approximal to each other. Then, in the 13th cycle, fatigue failure occurred, resulting in the end of the experiment. Owing to the increase of pore-water pressure, the effective stress decreased correspondingly, in addition to the load-bearing capacity of the specimen, so that when stress was applied to the upper limit of a setting, the specimen had entered the yielding stage, showing strain softening behaviour. The higher pore-water pressure hindered the closure of the primary fractures and the weak surfaces in the shale specimen during loading, but it promoted the reopening of the fractures and the generation of secondary cracks. This caused the continuous increase of plastic deformation, finally resulting in the failure of the shale. The results showed that when the pore-water pressure was 2, 6 and 10 MPa, the plastic deformation behaviour of the shale during the cyclic loading–unloading stages was similar. However, when it was set to 14 MPa, the shale softening was more significant, and broke down during the cyclic loading–unloading stages.

## 4. Analysis of deformation characteristics

### 4.1. Cyclic hardening and softening

Cyclic hardening and softening are important concepts when it comes to the delineation of the mechanical properties of rock. Under the controlled stress ranges, the cyclic hardening and softening of rock illustrate the decrease and the increase in axial strain amplitude [34]. In order to investigate the cyclic hardening and softening behaviour of the shale under different pore-water pressures and cyclic loading conditions, a detailed comparison analysis was carried out on the cyclic mean strain and strain amplitude. Cyclic mean strain [35] is

4.1 $${\bar{\varepsilon }}_{\;pm}=\frac{\varepsilon_{1,\max}+\varepsilon_{1,\min}}{2},$$

where ${\bar{\varepsilon }}_{\;pm}$ is the cyclic mean strain, *ɛ*_1,max_ is the strain value corresponding to the upper limit of stress *σ*_1,max_, and *ɛ*_1,min_ is the strain value corresponding to the lower limit of stress *σ*_1,min_. Strain amplitude is

4.2 $$\varepsilon_{a}=|\varepsilon_{1,\max}-\varepsilon_{1,\min}|.$$

**Figure 8.**
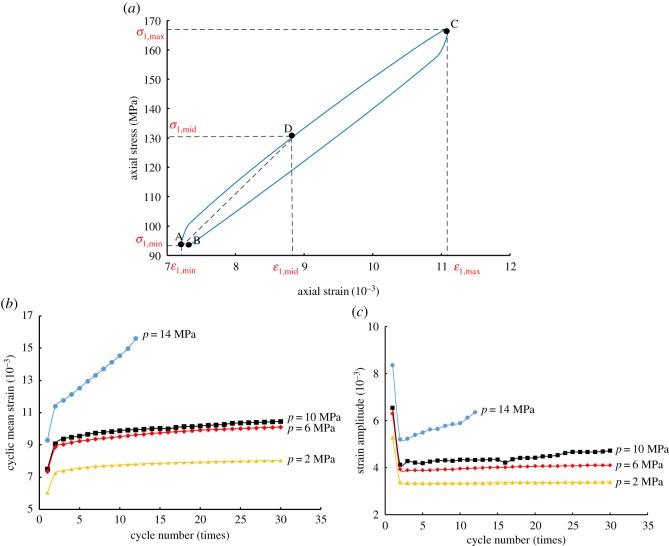
The curves of axial cyclic mean strain and strain amplitude under different pore-water pressures: (*a*) the curve of a single cycle; (*b*) the curve of cyclic mean strain; (*c*) the curve of strain amplitude.

The cyclic mean strain and strain amplitude under cyclic loading can be calculated from the strain parameters of the stress–strain curve, as shown in figure 8*a*. The cyclic mean strain under cyclic loading can be used to indicate irreversible (plastic) deformation, and the strain amplitude can be used to indicate elastic deformation [35].

As per the analysis results shown in figure 8*a*, the curves of the cyclic mean strain–cycle and the strain amplitude–cycle—based on the cycle number under different pore-water pressures—were obtained and compared. As shown in figure 8*b*, the initial strain of the axial cyclic mean strain exhibited an obvious upward trend when subjected to different pore-water pressures. When the pore-water pressure was set to 2, 6 and 10 MPa, the axial cyclic mean strain remained stable with the increase of the cycle number, but the axial cyclic mean strain tended to increase with the increase of the pore-water pressure. The cyclic mean strain at a pore-water pressure of 14 MPa increased rapidly with the increase of the cycle number and quickly reached the failure point. Furthermore, the strain was higher than that of the first three pore-water pressures, indicating that the cyclic mean strain was positively correlated with the pore-water pressure.

As shown in figure 8*c*, the initial axial strain amplitude experienced a significant decline under all pore-water pressures. When the pore-water pressures were 2, 6 and 10 MPa, the axial strain amplitude increased slowly with the increase of the cycle number, and then quickly reached stability within five cycles, whereas with increasing cycle numbers the strain amplitude shows a ‘V’ shape at a pore-water pressure of 14 MPa. Also, it is found that the axial strain amplitude was positively correlated with pore-water pressure. The cyclic softening phenomenon was not obvious under the pore-water pressures of 2, 6 and 10 MPa, whereas it became obvious when the pore-water pressure was increased to 14 MPa. Therefore, both cyclic mean strain and strain amplitude are positively correlated with the pore-water pressure.

### 4.2. Analysis of cyclic elastic constants

In the testing of the triaxial cyclic loading of shale, the elastic constants during the cyclic loading–unloading processes are usually calculated by the method introduced in *Specifications for Rock Tests in Water Conservancy and Hydroelectric Engineering* (SL264-2001) [36].

The calculation method of deformation modulus is as follows:

4.3 $$E_{50}=\frac{\sigma_{1,h}-\sigma_{1,\min}}{\varepsilon_{1,h}-\varepsilon_{1,\min}},$$

where *E*_50_ is the deformation modulus, *σ*_1,h_ is half of the maximum axial stress in each cycle, *σ*_1,min_ is the minimum axial stress in each cycle, *ɛ*_1,h_ is the axial strain corresponding to *σ*_1,h_, and *ɛ*_1,min_ is the axial strain corresponding to *σ*_1,min_.

However, the cyclic loading–unloading experiments carried out in this study have constant amplitude and fixed upper and lower limits. Meanwhile, *σ*_1,h_ and *σ*_1,min_ are constants here, and they do not vary with *ɛ*_1,h_ and *ɛ*_1,min_, so it is necessary to revise the existing formula.

As shown in figure 8*a*, points A and C correspond to the lower and upper stress limits of the cycle respectively, and point D corresponds to the intermediate stress. The required deformation modulus is equivalent to the slope of secant AD, and the formula is as follows:

4.4 $$\begin{array}{rl} {E^{\prime }}_{50} & =\frac{\sigma_{1,\mathrm{mid}}-\sigma_{1,\min}}{\varepsilon_{1,\mathrm{mid}}-\varepsilon_{1,\min}}\\ \sigma_{1,\mathrm{mid}} & =\frac{\sigma_{1,\max}+\sigma_{1,\min}}{2} \end{array}\}$$

where $E_{50}^{\prime }$ is the corrected deformation modulus, *σ*_1,mid_ is the intermediate stress of equal amplitude cycle, *ɛ*_1,mid_ is the strain corresponding to *σ*_1,mid_, and *σ*_1,max_ is the maximum axial stress in the cycle.

The elastic modulus can be calculated from the linear section of the stress–strain curve and the elastic modulus [36] can be calculated as follows:

4.5 $$E_{s}=\frac{\Delta \sigma }{\Delta \varepsilon },$$

where *E*_s_ is the elastic modulus, Δ*σ* is the stress difference of 10 MPa near *E*_s_, and Δ*ɛ* is the strain difference corresponding to Δ*σ*.

The cyclic elastic constants ($E_{50}^{\prime }$, *E*_s_) in each loading and unloading stage for each cycle were calculated based on the experimental results of different specimens under different pore-water pressures. In consonance with the calculations, the relationship between the cyclic elastic constants and the number of cycles of shale has been established.

**Figure 9.**
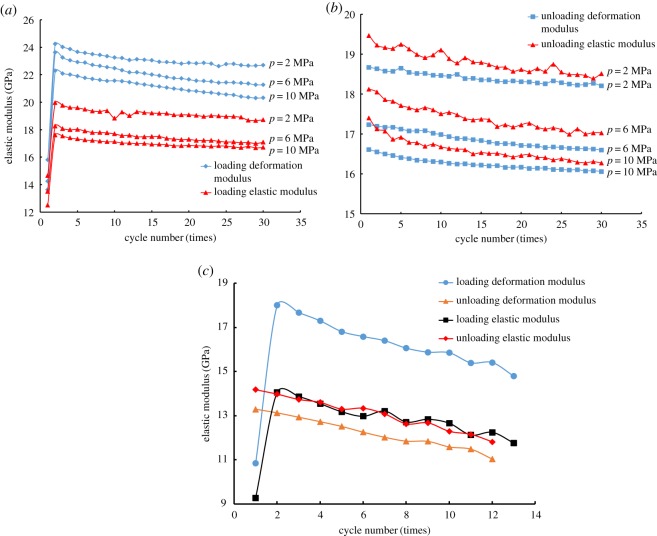
The cyclic elastic constants of shale under different pore-water pressure: (*a*) the loading elastic (deformation) modulus when the pore-water pressures are 2, 6 and 10 MPa; (*b*) the unloading elastic (deformation) modulus when the pore-water pressures are 2, 6 and 10 MPa; (*c*) the elastic modulus at a pore-water pressure of 14 MPa.

Firstly, the curves of elastic (deformation) modulus versus cyclic times were compared for the shale specimens under different pore-water pressures. It has been found that both the loading and unloading elastic modulus decreased with the increase of pore-water pressure, showing a negative correlation. As shown in figure 9*a*, when the pore-water pressures were 2, 6 and 10 MPa, both the loading elastic modulus and the loading deformation modulus initially increased sharply and then decreased slowly with the increase of cycle times. The natural fractures and micro-structural surfaces existing inside the shale did not close completely when the axial load was monotonically increased. This is due to the existence of pore-water pressure and the small load on the specimen. Then, with the continuous increase of axial load, the fractures and micro-fractures gradually closed. This resulted in a large plastic strain, so the loading elastic modulus and deformation modulus in the first cycle were low. Consequently, the calculated elastic modulus under different pore-water pressures was 12.54, 13.59 and 14.64 GPa, and the loading deformation modulus was 13.63, 14.24 and 15.80 GPa. After that, during the subsequent unloading process, some of the fractures were unable to fully reopen due to the friction between the micro-crack interfaces [15]; thus, the internal structure became denser, thereby increasing the overall stiffness of the shale. The calculated loading elastic modulus of the second cycle was 19.95, 18.26 and 17.61 GPa, and the loading deformation modulus was 24.24, 23.62 and 22.28 GPa, both of which increased significantly compared to those of the first cycle. Since then, as the number of cycles increased, fractures and micro-cracks in the shale gradually expanded. Because the pore-water pressure and the cyclic stress levels were not high, the accumulative process of plastic deformation was slow, so the calculated loading elastic (deformation) modulus also decreased slowly.

Figure 9*b* shows the unloading deformation modulus and elastic modulus of different specimens, which was different from the tendency of the elastic modulus, which would firstly increase and then decrease during the loading process. However, both the unloading elastic modulus and the deformation modulus appeared to decrease slowly, and the unloading elastic modulus showed some volatility.

As shown in figure 9*c*, when the pore-water pressure is increased to 14 MPa, both the loading elastic modulus and the unloading elastic modulus were significantly lower than those corresponding to the elastic modulus in figure 9*a*,*b*. As described above, after the loading–unloading process of the first cycle, the modulus of elasticity (deformation) increased rapidly, and the overall stiffness of the specimen also increased. During the unloading process, the higher pore-water pressure affected the friction between micro-cracks, and the plastic strain increased constantly. This resulted in a continuous decreasing of the elastic (deformation) modulus. With the increase of loading–unloading times, cracks and micro-cracks gradually expanded, converged and passed through to form macro-fractures. Furthermore, the internal structures were continuously adjusted locally, eventually resulting in the destruction of the specimens.

Comparing the cyclic elastic constants during the loading–unloading process, the loading deformation modulus was always greater than the loading elastic modulus, while the unloading elastic modulus was greater than the unloading deformation modulus. This corresponds to the rapid increase of axial strain during the loading process and the rapid decrease of axial strain during the unloading process, which is consistent with the previous analysis. Combined with the results of the whole calculation, although the cyclic elastic constants ($E_{50}^{\prime }$, *E*_s_) obtained by different calculation methods are different in size, the overall trend of change is consistent, which can reflect the change of the internal structure and the overall stiffness of rock. When the pore-water pressures were 2, 6 and 10 MPa, the loading elastic (deformation) modulus of the shale increased rapidly, then decreased slowly. However, when the pore-water pressure increased to 14 MPa, the elastic modulus of the specimen decreased dramatically, and the damage also occurred rapidly. The adjustment of the internal structure of the shale is notably evidenced by the elastic modulus.

## 5. Analysis of fracture characteristics

**Figure 10.**
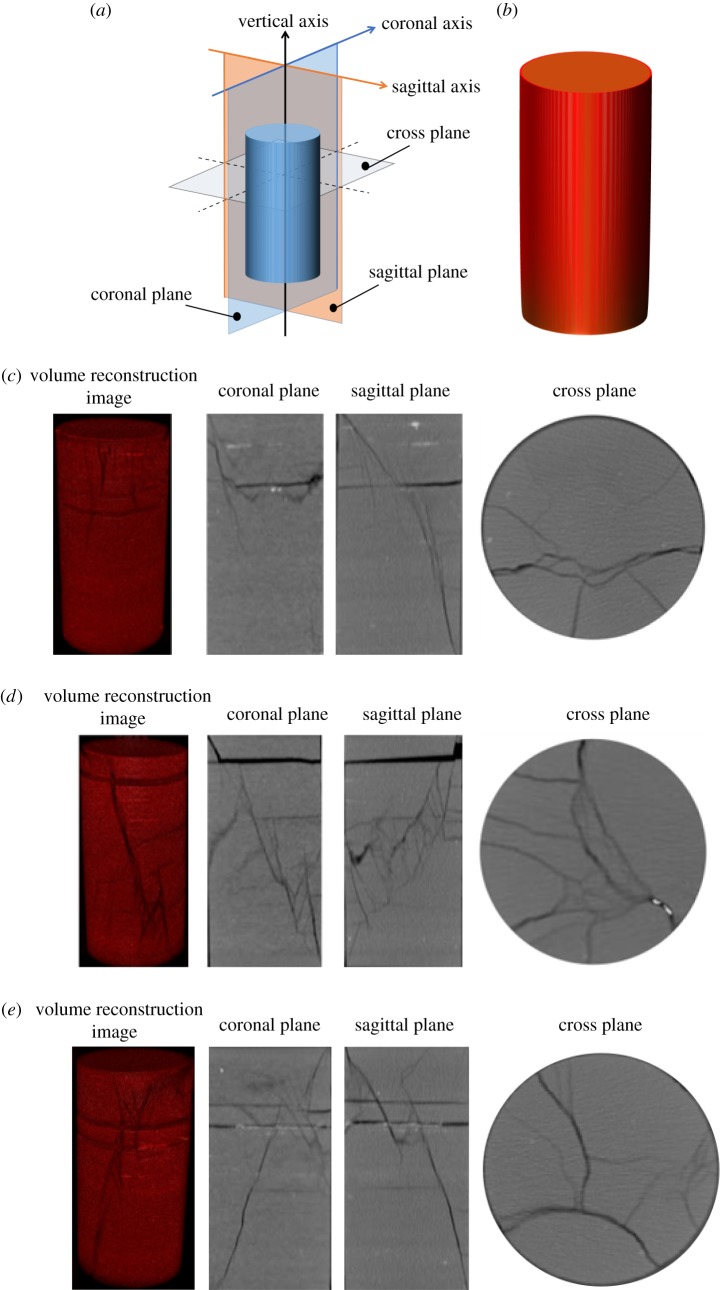
The computed tomography scan screenshots of shale under different pore-water pressures: (*a*) the diagrammatic sketch of coronal plane, sagittal plane and cross plane; (*b*) the volume reconstruction image of shale; (*c*) saturated shale under triaxial monotonic compression; (*d*) shale under triaxial cyclic loading with a pore-water pressure of 6 MPa; (*e*) shale under triaxial cyclic loading with a pore-water pressure of 14 MPa.

The damaged shale specimens were scanned by computed tomography after the experiment using different loading methods and different pore-water pressures. As shown in figure 10*a*,*b*, the coronal plane, sagittal plane and cross plane of the geometric centre in the specimen were respectively intercepted, as well as the external surface image of shale after volume reconstruction.

As shown in figure 10*c*, it was mainly shear failure that occurred in the saturated shale at a confining pressure of 20 MPa under triaxial monotonic compression. There was an obvious fracture plane of compression shear failure through multiple bedding planes in the shale. At the same time, a fracture along the bedding plane occurred on the upper part of the specimen. Because of the friction between the end face of the specimen and the indenter of the test machine, the lateral deformation of the end of the specimen was restrained, and the shear failure of the cross-bedding plane was formed under the larger lateral tension in the middle part of the specimen. In addition, after the saturation of dry shale, the internal bedding plane of the shale was weakened by the squeezing of pore water, and the connecting effect of the bedding plane was also weakened. This caused the rock specimens to crack along the bedding surface after being affected by shear failure.

According to figure 10*d*, when the pore-water pressure was 6 MPa, the failure mode of the shale under triaxial cyclic compression was primarily shear failure, from which the complex secondary fractures were derived. Axial load was continually added until the fracture occurred, for the specimen was not destroyed after the completed process of cyclic compression, indicating that the failure mode was compression shear failure. Cracks began to form at either end of the specimen, forming a shear crack through the bedding plane, which then split into several secondary cracks. This is an indication that the micro-cracks had enough time to initiate and expand under cyclic loading. At the same time, cracking along the bedding plane also occurred on the upper part of the shale caused by pore-water pressure.

As shown in figure 10*e*, when the pore-water pressure was 14 MPa, the failure mode of the specimens was also compression shear failure. Owing to higher pore-water pressure, the radial effective pressure and restraint capacity were reduced. As the shear stress on the rupture surface increased, shear failure of the specimen occurred. Additionally, some secondary cracks were generated close to the main shear cracks, due to the effect of axial cyclic loading. However, the number of cracks was not sizeable, as a result of the small the number of cycles applied for the test under a pore-water pressure of 14 MPa. Simultaneously, the larger pore-water pressure seriously weakened the shale bedding surface, causing the specimen to break into three parts along two parallel bedding planes.

The comparison shows that the failure mode of the shale was mainly compression shear failure, for both monotonic and cyclic loading tests, which is consistent with the existing experimental study [24]. In addition, axial cyclic loading caused secondary cracks in the specimen, and the larger the number of cycles, the more complex were the secondary cracks. Moreover, pore-water pressure weakens the shale bedding, causing it to crack under the influence of shear fracture surface; the greater the pore-water pressure, the more obvious this effect.

## 6. Conclusion

The shale softened after it was saturated with water, resulting in a significant reduction in failure strength at a larger axial strain. Furthermore, there was no pore-compaction process evident in the early stage of the triaxial compression tests.

A complete cyclic loading–unloading process can be divided into four stages under the coupling action of axial cyclic loading and pore-water pressure, which can be evidenced via the slow increasing and accelerated increasing of strain in the loading stage, as well as the slow decreasing and accelerated decreasing of strain in the unloading stage. In addition, the plastic deformation of the previous cycles was more prominent. This then decreased gradually but did not decrease to zero.

The axial plastic deformation behaviour was found to be similar when the pore-water pressures were set to 2, 6, and 10 MPa. Nevertheless, the shale softened notably, and fatigue damage occurred during the circulation process when the pore-water pressure was set to 14 MPa.

Axial cyclic mean strain and strain amplitude are positively correlated with pore-water pressure, while the elastic modulus is negatively correlated with pore-water pressure. As the cycle progresses, the trends in these parameters vary, indicating that the deformation and elastic characteristics of shale are controlled by pore-water pressure, together with cyclic loading. Between them, the effect of pore-water pressure is more significant.

In the triaxial compression tests, primarily shear failure occurred in the shale specimens. In addition, the axial cyclic loading caused the shale to generate complex secondary fractures; the specimens cracked along the bedding plane due to the effect of pore-water pressure.

## Supplementary Material

The original data of the experiments under pore-water pressure of 2MPa

## Supplementary Material

The original data of the experiments under pore-water pressure of 6MPa

## Supplementary Material

The original data of the experiments under pore-water pressure of 10MPa

## Supplementary Material

The original data of the experiments under pore-water pressure of 14MPa

## Permission to carry out fieldwork

Permission to collect shale was from the local government in Shuanghe Town, Changning County, Yibin City, Sichuan Province.

## Data accessibility

We have uploaded our original data of the experiments as electronic supplementary material along with the main manuscript.
